# Transgender people experience more discrimination and violence than cisgender lesbian, gay, or bisexual people: A multilevel analysis across 30 European countries

**DOI:** 10.1080/26895269.2024.2440856

**Published:** 2024-12-23

**Authors:** Jacob Evje, Sam Fluit, Tilmann von Soest

**Affiliations:** a CENSE – Norwegian Research Centre for Sexual Health, Department of Psychology, University of Oslo, Oslo, Norway; b PROMENTA Research Center, Department of Psychology, University of Oslo, Oslo, Norway; c Norwegian Social Research (NOVA), Oslo Metropolitan University, Oslo, Norway

**Keywords:** Discrimination, LGBTQ+ equality, multilevel analysis, trans rights, violence

## Abstract

**Introduction:**

Considering recent waves of transphobia, it is crucial to comprehensively research transgender people’s experiences with discrimination and violence. This study investigates the extent of discrimination and violence experienced by trans people and cisgender lesbian, gay and bisexual (LGB) people in Europe. We explore how these experiences vary across countries, how they are affected by a person’s disability and ethnic minority background, and whether experiences vary between trans women, trans men, and nonbinary people.

**Method:**

Using the 2020 EU LGBTI Survey II, we conducted multilevel analyses across 30 European countries on experiences of discrimination and violence among trans people and cisgender LGB people (*N* = 138,212). The 2019 Rainbow Europe Country Ranking was included as a country-level predictor.

**Results:**

Our findings demonstrated that trans people reported consistently more discrimination and violence than cisgender LGB people throughout Europe, irrespective of the countries’ rankings of LGBTQ+ rights. This difference was more pronounced among participants with ethnic minority backgrounds and disabilities. Results also varied across genders as trans women and nonbinary people reported more violence than trans men, whereas trans men and trans women both reported more discrimination than nonbinary people.

**Conclusions:**

This study provides compelling evidence of disproportionately negative experiences of trans people in Europe. The findings underscore the need for policy interventions and future studies addressing discrimination and violence faced by trans people.

Transgender people frequently face discrimination and harassment. On average, 60% of trans individuals across Europe reported discrimination because of their identity in the past year and 34% experienced physical or sexual attacks over the last five years (FRA, 2020). Discrimination and victimization have been linked to depression and suicidal ideation among trans individuals (Cunningham et al., 2022; Marzetti et al., 2024). Although LGBTQ+ rights vary greatly across European countries, sexual orientation and same-sex partnership rights tend to progress, whereas gender recognition laws and trans rights are stagnating or regressing in several countries (ILGA Europe, 2019). Given the current intensified wave of anti-trans mobilization (Grzebalska et al., 2017; Vincent et al., 2020), it is imperative to direct attention to addressing violence and discrimination faced by trans people compared to cisgender LGB people.

The academic literature has been criticized for generalizing queer experiences (Corrington et al., 2020; Worthen, 2013) and overlooking important differences in health risks by not examining people with different LGBTQ+ identities independently (Smalley et al., 2016). This gap is partly due to challenges in recruiting samples large enough to achieve sufficient statistical power for such analyses. Most studies on the experiences of trans people therefore adopt a qualitative approach and consider them separately from other LGBTQ+ populations (Bower-Brown et al., 2023; Dispenza et al., 2012). The few existing quantitative studies tend to present only trans experiences or compare them to the general population. This limits the insights into differences in experiences between trans people and cisgender LGB people and lacks comprehensive examinations across intersectional dimensions (Bower-Brown et al., 2023; Leonard & Mann, 2018; Kattari et al., 2017).

Addressing these gaps, this study aims to identify differences in experienced discrimination and violence between trans people and cisgender LGB people. In line with common conceptualizations in the field, we use “transgender” and “trans” as umbrella terms to include people whose assigned sex at birth does not align with their gender identity and sense of self (Aultman, 2014; Vincent et al., 2020). We include trans participants of any sexual orientation and cisgender lesbian, gay, and bisexual people. Further, we examine how these experiences are affected by disability status and ethnic minority identity, and how they vary between trans women, trans men, and nonbinary/gender diverse people. We use large-scale cross-national data from the 2020 EU LGBTI Survey II, with responses from more than 130,000 LGB and trans people across 30 European countries. These unique data give us the possibility to also study how differences in experiences vary across European countries and to what extent they are affected by the rules and regulations present in each country.

## Discrimination and violence against trans people

Several studies have documented the high prevalence of experiences with discrimination against transgender people, using qualitative methods (Dispenza et al., 2012; Papadaki & Ntiken, 2023), experimental field studies (Granberg et al., 2020), and quantitative analyses (Rodriguez et al., 2018). Trans people are also at increased risk of multiple types of violence, including sexual violence and domestic abuse, throughout their lives (Bachman & Gooch, 2018; Lombardi et al., 2001; Stotzer, 2009). Only one study directly compared the experiences of trans people to those of cisgender LGB people and found that trans people were more likely to experience discrimination and violence (Bayrakdar & King, 2023). However, their analyses were limited to Germany, Portugal, and the United Kingdom in 2014. Another study, using EU data from 2008, found that trans people were three times more likely to experience hate crimes (Turner & Whittle, 2012). Given the recent changes in social stigma from rising anti-trans movements, this research is becoming increasingly outdated (Vincent et al., 2020). To fill this research gap, we utilize recent data to provide a comprehensive overview of the current experiences of trans people and cisgender LGB people across 30 European countries.

## Intersectional and contextual perspectives on trans issues

Experiences with discrimination and violence likely differ among trans people. Based on intersectional approaches (Crenshaw, 2017), we study trans people from different countries, ethnic backgrounds, (dis)abilities, and gender identities.

Existing research indicates that levels of discrimination and violence experienced by trans people and cisgender LGB people vary across Europe (Bayrakdar & King, 2023; Turner & Whittle, 2012) due to differing national laws and policies (Bränström & Pachankis, 2021). Anti-discrimination and anti-violence policies vary substantially between European countries, and those protecting sexual orientation are more widespread than those protecting (trans)gender identity (ILGA Europe, 2019; TGEU, 2019). Initial research shows that country-level structural stigma negatively affects life satisfaction among sexual minorities across Europe (Bränström & Pachankis, 2023). However, no study has yet examined the relationship between national LGBTQ+ rights and experiences with discrimination and violence.

Queer people of color often face intersectional and cumulative discrimination because of racism within LGBTQ+ movements and anti-queer attitudes within ethnic groups (Cyrus, 2017). Research shows that anti-trans mobilizations target racialized (and especially Black) women and nonbinary people (Patel, 2017). Empirical studies show that trans people with ethnic minority backgrounds experience more discrimination compared to trans people with ethnic majority backgrounds (Kattari et al., 2017), but this issue has not yet been explored for experiences with violence. Moreover, there is a lack of research examining whether the gap between trans people’s and cisgender LGB people’s experiences with discrimination and violence might be larger within ethnic minority groups.

Due to societal ableism, trans individuals with disabilities can face additional challenges such as restricted capacity to express their gender identities, limited access to administrative processes, and more severe medical and legal gatekeeping (Baril et al., 2020; Riggs & Bartholomaeus, 2017). Indeed, some empirical findings indicate that trans people with disabilities experience higher levels of discrimination than trans people without disabilities (Kattari et al., 2017), and more violence and discrimination compared to cisgender LGB people with disabilities (Leonard & Mann, 2018). However, we still need intersectional studies on disabled trans experiences with larger samples and more demographic diversity.

Moreover, differences among trans people with various gender identities are not well studied, as nonbinary identities are often omitted in research or trans people are studied as one uniform group (Bower-Brown et al., 2023). Empirical findings indicate that trans women and nonbinary people experience more harassment and discrimination than trans men (Devís-Devís et al., 2022; Harrison et al., 2012; Turner & Whittle, 2012). However, empirical findings on experiences with discrimination and violence among trans people with various gender identities have been limited in scope and derive mostly from the United States. As such, we compare trans people and cisgender LGB people in 30 countries in a wide array of sociopolitical contexts taking into account their ethnic backgrounds, (dis)ability status, and gender identity.

## The present study

To expand the limited amount of quantitative research on the experiences of trans people, we utilize recent data with a large sample size and demographic diversity from 30 European countries to investigate the differences between trans people’s and cisgender LGB people’s experiences with discrimination and violence. The following research questions are addressed: To what extent do trans people and cisgender LGB people experience different levels of discrimination and violence? Do these differences vary across countries based on their levels of LGBTQ+ rights? Are these differences more pronounced for participants with ethnic minority backgrounds and disabilities? To what extent does exposure to discrimination and violence vary between trans women, trans men, and nonbinary participants?

First, based on the existing body of research on this topic, we expect that trans participants will report more discrimination and more violence than cisgender LGB people. Second, regarding country differences, we expect that higher national LGBTQ+ rights will be associated with more positive experiences for participants, and we will explore whether this relationship holds equally for trans people and cisgender LGB people. Third, we expect that differences between trans people and cisgender LGB people will be more pronounced for people with ethnic minority backgrounds and disabilities. Lastly, we expect that trans women and nonbinary people will report more negative outcomes than trans men.

## Method

### Procedure

The EU LGBTI Survey II (FRA, 2020), collected in 2019, is the largest cross-national survey on the experiences of lesbian, gay, bisexual, trans and intersex (LGBTI) people across Europe. Deployed as an online, anonymous, opt-in survey, participants were recruited *via* both online and offline channels in 30 countries. Participants completed the questionnaire in their native language (for more information on the translation process, see FRA, 2020). The sample consisted of self-identified LGBTI people aged 15 or older, who had, at the time of participation, lived in an EU Member State, the United Kingdom, North Macedonia, or Serbia for at least one year, irrespective of citizenship or residency status. As intersex respondents did not fall within the scope of this study, we excluded 1,587 intersex individuals leaving a total number of 138,212 participants.

### Measures

#### Trans (versus cisgender LGB)

Aligning with previous methodologies (Bayrakdar & King, 2023), a dummy variable for trans was constructed (0 = cisgender LGB, 1 = trans). Several questions regarding participants’ gender and sexuality were used in the construction of the variable. The question “Are/were you a trans person?” was used to determine whether participants identified as transgender or cisgender. The question “Which group best matches your sexual orientation?” was then used to categorize cisgender people as either lesbian, gay, or bisexual, who were then grouped as cisgender LGB people. The question “How would you describe your current gender identity?” was used to categorize cisgender participants as either cisgender women or cisgender men, and trans participants as either transgender women, transgender men, or nonbinary/gender diverse (including all participants who identified as neither a man nor a woman).

#### Discrimination

When assessing discrimination, we created a sum score representing the number of areas of life where participants experienced discrimination. Participants were asked: “During the last 12 months, have you personally felt discriminated against because of being [RESPONDENT CATEGORY] in any of the following situations?”, with response options of *Yes*, *No*, *Haven’t done this*, and *Don’t know*. These options were provided for seven distinct domains: “when looking for a job”, “at work”, “when looking for a house or apartment to rent or buy”, “by healthcare or social services personnel”, “by school/university personnel”, “at a café, restaurant, bar or nightclub”, and “at a shop”. We then aggregated the *yes* responses to form the discrimination variable. Thus, this variable represents the breadth of discrimination experiences, with higher scores (ranging from 0 to 7) indicating exposure to discrimination across more domains, independent of the frequency of occurrences within each domain.

#### Violence

Experiences of violence were assessed by the item “In the last 5 years, how many times have you been physically or sexually attacked at home or elsewhere (street, on public transport, at your workplace, etc.) for any reason?” Responses included *never* (0), *once* (1), *twice* (2), *3-5 times* (3), *6-10 times* (4), *more than 10 times* (5), and *all the time* (6). The responses *Not sure* and *Prefer not to say* were treated as missing values. A higher score on this variable indicated more instances of exposure to physical or sexual violence.

#### Country and national LGBTQ+ rights

Participants selected their current country of residence from a drop-down menu. As an indicator of differences in overall LGBTQ+ rights between countries, we used scores from the Rainbow Europe Country Ranking (ILGA Europe, 2019). These scores range from 0% to 100% and compare countries’ legal standards with their European neighbors. The index is constructed by evaluating 72 types of laws and policies within seven categories; equality and nondiscrimination, family, hate crime and hate speech, legal gender recognition, intersex bodily integrity, civil society space, and asylum. Low LGBTQ+ rights scores indicate that a country has substantial violations of human rights and significant discrimination on the basis of sexual and romantic orientation or activity, gender identity, and gender expression. In contrast, high LGBTQ+ rights scores indicate that a country respects human rights and ensures equality through recognition and specific protection of LGBTQ+ people under the law in all areas of society (ILGA Europe, 2019).

#### Disability and ethnic minority status

Dummy variables were constructed for ethnicity (0 = ethnic majority, 1 = ethnic minority), and disability (0 = no disability, 1 = disability). To determine these variables, participants self-reported additional minority status by answering the following question, “In the country where you live, do you consider yourself to be part of any of the following, other than LGBTI?”. Options included *An ethnic minority (including those of migrant background)* and *A minority in terms of disability*.

#### Covariates

We included participants’ age and financial situation as covariates, as discrimination and violence may differ based on people’s life stages and economic resources (Potter et al., 2019). To ensure the anonymity of the respondents, the dataset included aggregated age categories ranging from 1 (15-17 years old) to 11 (65+ years old). As an approximation of financial situation, participants were asked “Thinking of your household’s total income, is your household able to make ends meet?”. Responses ranged from *With great difficulty* (1) to *Very easily* (6). Response options *Prefer not to say* and *Don’t know* were treated as missing values.

## Analysis

Using R version 4.2.2 (R Core Team, 2022), we estimated multiple multilevel models for discrimination and violence, separately. Multilevel analyses extend traditional regression models by allowing for the analysis of data that are organized at more than one level, accounting for participants being clustered within countries (Snijders, 1999; Bickel, 2007). Like traditional regression models, multilevel regression analyses enable the inclusion of several explanatory variables simultaneously, allowing us to assess the unique predictive value of one variable while accounting for the influence of other variables included in the model. In our study, the outcome variable for discrimination was operationalized as a sum score of 7 dichotomous ordinal variables, each indicating the presence or absence of discrimination in specific areas. Such discrete sum scores can be viewed as a robust ordinal approximation of a continuous latent variable (Sijtsma et al., 2024). For violence, we used a seven-point scale where each response option represents increasing increments of violence experienced. According to the methodological literature, such measures can be used as an ordinal approximation of a continuous variable and parametric tests are sufficiently robust to yield largely unbiased estimations (Norman, 2010; Sullivan & Artino, 2013). Thus, multilevel linear regression models were considered appropriate for our aims.

Following the methodological recommendations for multilevel modeling (Hox, 2010; Moerbeek et al., 2017; Finch et al., 2019), the variables were introduced stepwise in multiple models and the data were analyzed at the individual level (level 1) and the country level (level 2) simultaneously. In each model, new variables were added in addition to previously added variables. The predictors of trans identity (versus cisgender LGB), ethnic minority (versus ethnic majority), disability (versus no disability), age, financial situation, national LGBTQ+ rights and interaction terms were added as fixed effects, while the grouping variable of country of residence was entered as a random effect. Maximum likelihood estimation was used for all models, utilizing the lme4 package (Bates et al., 2015). The lmerTest package (Kuznetsova et al., 2017) was used to estimate confidence intervals and p-values of fixed and random effects. National LGBTQ+ rights, age, and financial situation were grand-mean-centered to increase the interpretability of the models. To compare model fit, the Akaike information criterion (AIC) of each model was assessed, in which lower scores indicated a better fit (Heck et al., 2011).

All questions used in the current study were forced-choice items where respondents were required to provide a response to be able to proceed to the next question. For the measure of violence, 380 participants (0.3% of the sample) responded with *Not sure* or *Prefer not to say,* and these responses were treated as missing values. Similarly, for the measure of financial status, we coded as missing the responses for the 395 participants (0.3% of the sample) who selected *Prefer not to say* or *Don’t know*. The dataset did not contain any other missing values for any variable at either the individual or country level. Little’s test of missing completely at random (Little, 1988) showed that data were not completely at random (*p* < .001). However, due to the minimal amount of missing data, these were handled by listwise deletion.

## Ethics

This project has been ethically evaluated and approved by the Department of Psychology’s internal research ethics committee, University of Oslo (ref. number: 21730390). Approval to use the data was given by the European Union Agency for Fundamental Rights after agreeing to the obligations to maintain data privacy and the required ethical standards of data management. Survey participants were informed of the types of data that were collected, how the data would be processed and used, who could have access to data, and what security measures would be taken to safeguard personal data. All participants provided informed consent before study participation.

## Results

### Participants

Of the total 138,212 participants, 118,543 (85.8%) were cisgender LGB, of which 19.2% were lesbian women, 49.7% gay men, and 31.2% bisexual individuals. Of all participants, 19,669 were trans, of which 19.8% were trans women, 27.7% trans men, and 52.4% nonbinary/gender diverse people. Most participants (64.1%) were between 15 and 29 years old. A total of 7.4% of participants reported belonging to an ethnic minority, while 4.9% reported having a disability. Concerning financial situation, 34.6% indicated that their households had difficulties to make ends meet. Detailed information on participant demographics is presented in Table 1.

**Table 1.** Demographic information by cisgender LGB, trans, and all participants.

	Cisgender LGB	Trans	All
	*n* = 118,543	*n* = 19,669	*n* = 138,212
Gender identity, *n* (%)			
Woman	49,924 (42.1)	3,903 (19.8)	53,827 (38.9)
Man	68,619 (57.9)	5,453 (27.7)	74,072 (53.6)
Nonbinary/gender diverse		10,313 (52.4)	10,313 (7.5)
Sexual orientation, *n* (%)			
Lesbian	22,707 (19.2)	3,256 (16.6)	25,963 (18.8)
Gay	58,908 (49.7)	2,551 (13.0)	61,459 (44.5)
Bisexual	36,928 (31.2)	7,358 (37.4)	44,286 (32.0)
Heterosexual		1,667 (8.5)	1,667 (1.2)
Other		4,746 (24.1)	4,746 (3.4)
Don’t know		91 (0.5)	91 (0.1)
Ethnicity, *n* (%)			
Ethnic majority	109,793 (92.6)	18,227 (92.7)	128,020 (92.6)
Ethnic minority	8,750 (7.4)	1,442 (7.3)	10,192 (7.4)
Disability, *n* (%)			
Not disabled	114,296 (96.4)	16,988 (86.4)	131,284 (95.0)
Disabled	4,147 (3.5)	2,681 (13.6)	6,828 (4.9)
Age group, *n* (%)			
15-17	14,775 (12.5)	3,634 (18.5)	18,409 (13.3)
18-24	40,830 (34.4)	8,442 (42.9)	49,272 (35.6)
25-29	18,094 (15.3)	2,918 (14.8)	21,012 (15.2)
30-39	22,511 (19.0)	2,501 (12.7)	25,012 (18.1)
40+	22,333(18.8)	2,174 (11.1)	24,507 (17.7)
Financial situation, *n* (%)			
No financial difficulties	76,740 (64.7)	10,436 (53.1)	87,176 (63.1)
Financial difficulties	41,488 (35.0)	9,153 (46.5)	50,641 (34.6)

The countries with the largest number of participants were Spain (14.5%), Germany (11.5%), Poland (9.8%), France (9.6%), and the United Kingdom (8.8%). The average national LGBTQ+ rights score for all countries included was 47.56 (out of 100), with a standard deviation (*SD*) of 18.50. As such, the average was lower than the midpoint of the scale and there was a substantive variability in equality and justice for LGBTQ+ people across European countries. Malta achieved the highest score across Europe, with a value of 90.0, indicating near-total equality under the law, reflected by Malta’s policies allowing for marriage and adoption for same-sex couples, with self-determination, legal gender recognition, and state-funded gender-affirming care for trans people. In contrast, North Macedonia had the lowest score with a value of 11.0, reflecting a lack of lack basic protections against discrimination and hate-motivated violence for LGBTQ+ people, as well as inadequate access to necessary medical care for this group. For a full list of the number of participants and LGBTQ+ rights scores per country, see Table 2.

**Table 2.** Number of participants and LGBTQ+ rights score per country.

Country	n (%)	LGBTQ+ rights score
Malta	784 (0.6)	90
Belgium	2,653 (1.9)	73
Luxembourg	358 (.3)	70
Finland	4,682 (3.4)	69
Denmark	2,225 (1.6)	68
Portugal	4,221 (3.1)	66
United Kingdom	12,144 (8.8)	66
France	13,332 (9.6)	63
Sweden	2,478 (1.8)	62
Spain	20,066 (14.5)	61
Austria	2,292 (1.7)	50
Netherlands	3,853 (2.8)	50
Greece	4,426 (3.2)	49
Croatia	1,071 (0.8)	47
Germany	15,959 (11.5)	47
Ireland	2,362 (1.7)	47
Hungary	4,016 (2.9)	41
Slovenia	626 (0.5)	40
Estonia	1,124 (0.8)	35
Slovakia	2,883 (2.1)	30
Serbia	1,640 (1.2)	28
Czech Republic	3,525 (2.6)	26
Cyprus	613 (0.4)	23
Lithuania	13,66 (1.0)	23
Italy	9,640 (7.0)	22
Romania	3,134 (2.3)	21
Bulgaria	1,876 (1.4)	20
Poland	13,543 (9.8)	18
Latvia	737 (0.5)	17
North Macedonia	583 (0.4)	11

### Descriptive statistics

Means, *SD*s, and intercorrelations for all study variables are displayed in Table 3. χ*^2^* tests were performed with Bonferroni corrections to account for multiple testing. For tests to be significant at *p* < .001, the corrected significance level was therefore *p* < .000063. Trans people reported significantly higher levels of discrimination, relative to cisgender (*M*_trans_ = 1.42, *M*_cisgender LGB_ = 0.77, χ*^2^* (7) = 2,233.97, *p* < .000063). More trans participants (58%) than cisgender LGB participants (40%) reported discrimination in at least one area of life during the past year (χ*^2^* (1) = 2,154.63, *p* < .000063). Moreover, trans people reported significantly higher levels of violence during the last five years (*M*_trans_ = 1.00, *M*_cisgender LGB_ = 0.56, χ*^2^* (6) = 4251.44, *p* < .000063). Additionally, more trans participants (40%) than cisgender LGB participants (25%) had been attacked at least once (χ*^2^* (1) = 1783.79, *p* < .000063), and more trans participants (5%) than cisgender LGB participants (2%) had been attacked more than 10 times (χ*^2^* (1) = 571.70, *p* < .000063).

**Table 3.** Means, standard deviations, and correlations of variables under study.

	Variables	Mean	SD	1	2	3	4	5	6	7
1	Trans (vs LGB)	14.2%								
2	Discrimination	0.86	1.3	.17***						
3	Violence	0.62	1.23	.13***	.31***					
4	National LGBTQ+ rights	47.13	18.55	.07***	−0.00	−0.05***				
5	Ethnic minority	7.4%		−0.00	.05***	.04***	.05***			
6	Disability	4.9%		.16***	.09***	.08***	.07***	.04***		
7	Age	3.43	2.25	−0.10***	−0.09***	−0.15***	.12***	−0.02***	−0.00	
8	Financial situation	3.91	1.27	−0.10***	−0.18***	−0.15***	.03***	−0.03***	−0.09***	.07***

*Note*: For Violence, *N* = 137,832. For financial situation, *N* = 137,817. For all other variables, *N* = 138,212.

* *p*<.05, **p<.01, ***p<.001. The age variable refers to age categories from 1 (15-17 years old) to 11 (65+ years old).

Additional χ*^2^* tests were performed for each of the seven areas of discrimination. Significant differences were found between trans people and cisgender LGB people in all areas (*p* < .000063). Specifically, discrimination against trans people was more prevalent when looking for a job (trans = 12.7%, cisgender LGB = 3.5%), at work (trans = 18.3%, cisgender LGB = 13.3%), when buying or renting a house or apartment (trans = 6.7%, cisgender LGB = 5.0%), when accessing healthcare or social services (trans = 27.6%, cisgender LGB = 9.7%), at school or university (trans = 26.8%, cisgender LGB = 14.3%), at cafés, restaurants, or nightclubs (trans = 25.1%, cisgender LGB = 21.0%), and at shops (trans = 24.4%, cisgender LGB = 10.1%). In sum, trans participants reported substantially more discrimination and violence compared to cisgender LGB participants across all measures.

Further analyses showed that trans participants were proportionately more likely to report having a disability than cisgender LGB participants (χ*^2^* (1) = 3685.8, *p* < .000063), while the two groups did not significantly differ according to ethnic minority status (χ*^2^* (1) = 0.06, *p* = .815). Moreover, on average, trans participants in our sample were younger than cisgender LGB participants (χ*^2^* (10) = 1770.1, *p* < .000063) and had more financial difficulties (χ*^2^* (5) = 1503.7, *p* < .000063).

### Multilevel analyses

The results of the multilevel analyses are displayed in separate tables for discrimination (Table 4) and violence (Table 5). First, an intercept-only model, without any predictors, was estimated to calculate the intraclass correlation coefficient (ICC). The ICC indicated that 0.01% of the variation in discrimination, and 0.04% of the variation in violence was due to country-level effects. The country variance for both discrimination and violence was significant (*p* < .001).

**Table 4.** Multilevel analysis predicting discrimination.

	Model 1	Model 2	Model 3	Model 4	Model 5	Model 6	Model 7	Model 8
Fixed effects	Est. (SE)	Est. (SE)	Est. (SE)	Est. (SE)	Est. (SE)	Est. (SE)	Est. (SE)	Est. (SE)
Intercept	0.84***(0.02)	0.75***(0.03)	0.73***(0.02)	0.73***(0.02)	0.73***(0.02)	0.73***(0.02)	0.73***(0.02)	1.38***(0.04)
Trans		0.67***(0.01)	0.55***(0.01)	0.51***(0.03)	0.52***(0.02)	0.54***(0.01)	0.54***(0.01)	
Ethnic minority			0.19***(0.01)	0.19***(0.01)	0.19***(0.01)	0.17***(0.01)	0.19***(0.01)	0.27***(0.04)
Disability			0.29***(0.02)	0.29***(0.02)	0.29***(0.02)	0.29***(0.02)	0.24***(0.02)	0.36***(0.03)
Age			−0.04***(0.00)	−0.04***(0.00)	−0.04***(0.00)	−0.04***(0.00)	−0.04***(0.00)	−0.03***(0.01)
Financial situation			−0.16***(0.00)	−0.16***(0.00)	−0.16***(0.00)	−0.16***(0.00)	−0.16***(0.00)	−0.24***(0.01)
National LGBTQ+ rights				−0.00*(0.00)	−0.00*(0.00)	−0.00(0.00)	−0.00(.00)	−0.00(0.00)
Trans x National LGBTQ+ rights					0.00(0.00)			
Trans x Ethnic minority						0.11**(0.04)		
Trans x Disability							0.14***(0.03)	
Trans woman								−0.05(0.04)
Nonbinary								−0.32***(0.03)
**Random Effects**	Var.	Var.	Var.	Var.	Var.	Var.	Var.	Var.
Country	.02	.02	.01	.02	.02	.01	.01	.02
Residual	1.69	1.64	1.58	1.58	1.58	1.58	1.58	2.54
AIC	464739.9	460361.6	454309.1	454142.8	454142.7	454300.5	454292.7	73920.0
N	138,212	138,212	137,817	137,817	137,817	137,817	137,817	195,89

*Note:* Includes estimated fixed effects, standard errors for the estimate, variances for the random effects, Akaike Information Criterion (AIC), and the number of responses for each model. All regression coefficients are unstandardized.

*p<.05, **p<.01, ***p<.001.

**Table 5.** Multilevel analysis predicting violence.

	Model 1	Model 2	Model 3	Model 4	Model 5	Model 6	Model 7	Model 8
Fixed effects	Est. (SE)	Est. (SE)	Est. (SE)	Est. (SE)	Est. (SE)	Est. (SE)	Est. (SE)	Est. (SE)
Intercept	0.60***(0.03)	0.54***(0.03)	0.53***(0.03)	0.53***(0.03)	0.53***(0.03)	0.53***(0.03)	0.53***(0.03)	0.71***(0.04)
Trans		0.44***(0.01)	0.31***(0.01)	0.31***(0.02)	0.31***(0.02)	0.29***(0.01)	0.30***(0.01)	
Ethnic minority			0.14***(0.01)	0.14***(0.01)	0.14***(0.01)	0.11***(0.01)	0.14***(0.01)	0.32***(0.04)
Disability			0.32***(0.02)	0.32***(0.02)	0.32***(0.02)	0.32***(0.02)	0.3***(0.02)	0.35***(0.03)
Age			−0.07***(0.00)	−0.07***(0.00)	−0.07***(0.00)	−0.07***(0.00)	−0.07***(0.00)	−0.06***(0.01)
Financial situation			−0.12***(0.00)	−0.12***(0.00)	−0.12***(0.00)	−0.12***(0.00)	−0.12***(0.00)	−0.18***(0.01)
National LGBTQ+ rights				−0.00(0.00)	−0.00(0.00)	−0.00(0.00)	−0.00(0.00)	−0.00*(0.00)
Trans x National LGBTQ+ rights					−0.00*(0.00)			
Trans x Ethnic minority						.023***(0.04)		
Trans x Disability							0.07***(0.03)	
Trans woman								0.15***(0.03)
Nonbinary								0.16***(0.03)
**Random Effects**	Var.	Var.	Var.	Var.	Var.	Var.	Var.	Var.
Country	.03	.03	.02	.02	.02	.02	.02	.03
Residual	1.48	1.46	1.40	1.41	1.40	1.40	1.40	2.16
AIC	445286.7	443151.0	436378.9	436331.3	436329.0	436337.7	436377.0	70457.5
N	137,832	137,832	137,441	137,441	137,441	137,441	137,441	195,00

*Note:* Includes estimated fixed effects, standard errors for the estimate, variances for the random effects, Akaike Information Criterion (AIC), and the number of responses for each model. All regression coefficients are unstandardized.

*p<.05, **p<.01, ***p<.001.

The overall differences between trans people’s and cisgender LGB people’s experiences were tested by including the trans variable as a predictor in Model 2. We found that, as compared to cisgender LGB people, being trans positively and significantly predicted experiencing discrimination (*b* = 0.67, *SE* = 0.01, *p* < .001). This result held when controlling for ethnic minority status, disability, age, and financial situation in Model 3 (*b =* 0.55, SE = 0.01, *p* < .001). We found similar results for violence, when compared to cisgender LGB people, being trans positively and significantly predicted experiencing violence (*b* = 0.44*, SE* = 0.01*, p* < .001). This also held in Model 3 (*b =* 0.31, *SE =* 0.01, *p* < .001). Thus, trans people reported substantially more discrimination and violence than cisgender LGB people, even after controlling for ethnic minority status, disability, age, and financial situation.

**Figure 1.**
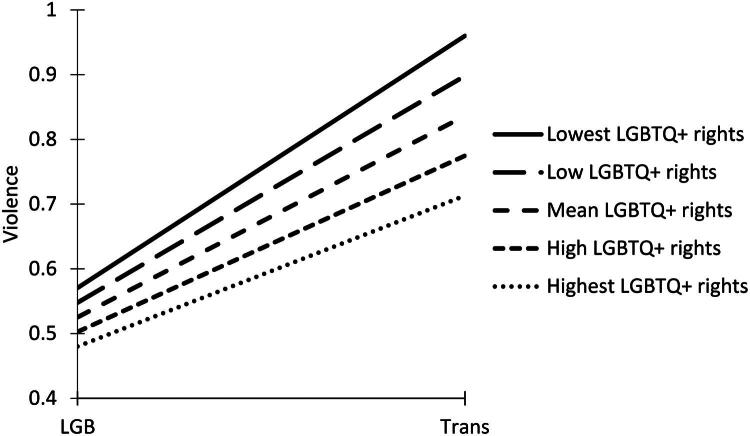
The association between national LGBTQ+ rights and violence against cisgender LGB people and trans people.

In Models 4 and 5, we explored how the differences between trans people and cisgender LGB people varied across countries. In Model 4, we added the national LGBTQ+ rights variable and random slopes which allowed associations between the trans predictor and outcomes to vary across countries. In this model, national LGBTQ+ rights were negatively and significantly associated with discrimination (*b* = −0.002, *SE* = 0.001, *p* = .023). In Model 5, we added an interaction term between the trans variable and the national LGBTQ+ rights variable. Here, the main effect of national LGBTQ+ rights remained significant (*p* = .041), but the cross-level interaction term (*b =* 0.002, *SE* = 0.001, *p* = .143) was not significantly associated with discrimination. Overall, this indicated that participants living in countries with higher national LGBTQ+ rights felt somewhat less discriminated against. National LGBTQ+ rights were not significantly associated with violence (*b =* −0.001, *SE* = 0.001, *p* = .329) in any of the models. However, the cross-level interaction term between the trans variable and the national LGBTQ+ rights variable was negatively and significantly associated with violence (*b =* −0.002, *SE* = 0.001, *p* = .042). Thus, lower LGBTQ+ rights were associated with slightly larger differences in levels of violence against trans people and cisgender LGB people (see Figure 1).

**Figure 2.**
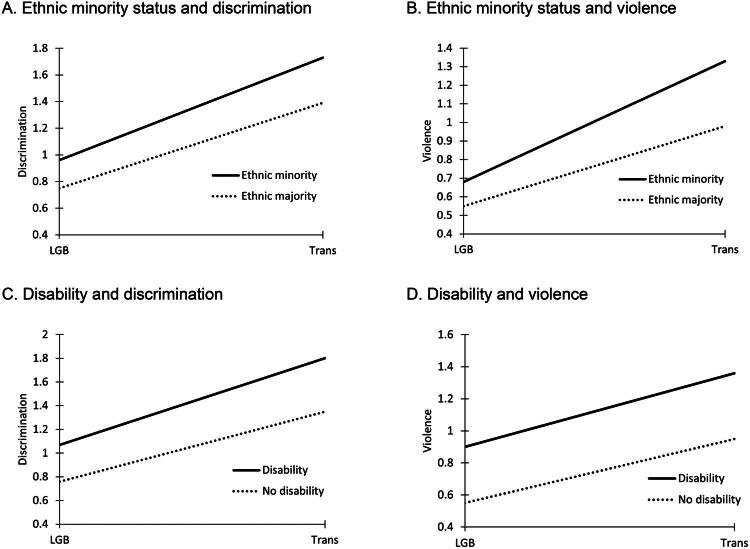
The association of disability and ethnic minority background with discrimination and violence against cisgender LGB people and trans people.

The associations of being an ethnic minority and having a disability with trans and LGB people’s experiences were tested in Models 6 and 7. In Model 6, an interaction term between the trans variable and the ethnic minority variable was added. In this model, the interaction term between being trans and being an ethnic minority was positively and significantly associated with discrimination (*b =* 0.11, *SE* = 0.04, *p* = .002) and violence (*b =* 0.23, *SE* = 0.04, *p* < .001). The main effects of being an ethnic minority also positively and significantly predicted discrimination (*b =* 0.17, *SE* = 0.01, *p* < .001) and violence (*b =* 0.11, *SE* = 0.01, *p* < .001). These findings indicated that participants with ethnic minority backgrounds, as compared to participants with ethnic majority backgrounds, experienced more negative outcomes, particularly those who were also trans (see Figure 2, Panels A & B).

In Model 7, an interaction term between the trans variable and the disability variable was added. Similarly, the interaction between being trans and having a disability was positively and significantly associated with discrimination (*b =* 0.14, *SE* = 0.03, *p* < .001), and violence (*b =* 0.07, *SE* = 0.03, *p* < .001). The main effects of having a disability also positively and significantly predicted discrimination (*b =* 0.24, *SE* = 0.02, *p* < .001), and violence (*b =* 0.30, *SE* = 0.01, *p* < .001). The results thus show that participants with disabilities, as compared to participants without disabilities, experienced more negative outcomes, particularly those who were also trans (see Figure 2, Panels C & D).

When studying differences between trans women, trans men, and nonbinary people in Model 8, cisgender LGB participants were excluded from the analyses. In this model, the trans variable was replaced with two dummy variables, contrasting trans women and nonbinary people with trans men, while covariates remained the same. Compared to trans men, being a trans woman was not significantly associated with discrimination (*b =* −0.05, *SE* = 0.04, *p* = .139), suggesting that trans women and trans men felt, on average, discriminated against in an equal number of areas of life. However, being a trans woman significantly predicted violence (*b =* 0.15, *SE* = 0.03, *p* < .001), thereby indicating that trans women experienced more violence than trans men. Last, compared to trans men, being nonbinary was negatively and significantly associated with discrimination (*b =* −0.32, *SE* = 0.03, *p* < .001) and positively associated with violence (*b =* 0.16, *SE* = 0.03, *p* < .001). This indicated that nonbinary people felt discriminated against in fewer areas of life but were subjected to more violence than trans men.

## Discussion

In this study, we aimed to examine differences between trans people’s and cisgender LGB people’s experiences with discrimination and violence. Moreover, we explored how these experiences varied across European countries, how they were affected by a person’s disability and ethnic minority identity, and how they varied among trans people with different gender identities. The results showed that trans people experienced more discrimination and violence than cisgender LGB people. These differences were consistent throughout European countries, indicating that these inequalities persist even in countries with high levels of LGBTQ+ rights. Further, lower LGBTQ+ rights were associated with more discrimination for all participants and slightly larger differences in levels of violence against trans people and cisgender LGB people. Participants with ethnic minority backgrounds and disabilities generally experienced more negative outcomes, particularly those who were also trans. Trans women and nonbinary people experienced more violence than trans men. In contrast, trans men and trans women reported similar levels of discrimination, whereas nonbinary people reported discrimination in fewer areas.

### Differences between trans and LGB experiences

The findings indicate that trans people face discrimination in more domains of life and experience violence more frequently compared to cisgender LGB people. This is in accordance with the findings of Bayrakdar and King (2023), who compared trans people’s and cisgender LGB people’s experiences in Germany, Portugal, and the United Kingdom. The present study expands on these findings by investigating many countries with varying political climates. We also used recent data to capture the diverse experiences of trans people and cisgender LGB people in Europe during a time of increased hostility. Additionally, previous research utilized dichotomous measures only indicating the presence of discrimination and violence. In our study, we assessed the frequency of violence reported and the number of life areas where discrimination was experienced, adding further nuance to previous studies in this field.

### Country differences

Our results showed rather small differences in trans people’s and cisgender LGB people’s experiences between countries. Moreover, we found a weak relationship between national LGBTQ+ rights and discrimination. Consistent with prior research (Bayrakdar & King, 2023; Turner & Whittle, 2012), participants living in countries with higher national LGBTQ+ rights reported slightly lower levels of being discriminated against. Interestingly, we found no main effect of LGBTQ+ rights on experiences with violence. However, countries with lower LGBTQ+ rights showed somewhat greater differences in levels of violence against trans than cisgender LGB people—a finding not reported in previous studies.

### Disability and ethnic minority status

Among both trans people and cisgender LGB people, having ethnic minority backgrounds and disabilities was related to experiencing higher levels of anti-queer discrimination and violence. This heightened risk may be attributed to greater visibility and non-normative status, as well as the cumulative negative stereotypes and attitudes that these intersectionally marginalized groups face (Cyrus, 2017; McRuer, 2016). Importantly, we found that this effect was more pronounced among trans participants than cisgender LGB participants. This difference may arise from anti-trans ideologies that rely on narratives that dehumanize ethnic minorities and stigmatize disability (Hsu, 2022).

Our findings align with previous research showing that among people with disabilities, trans people experience more discrimination and violence than cisgender LGB people (Leonard & Mann, 2018). However, our study is the first to examine this difference in discrimination against trans people and cisgender LGB people specifically within ethnic minority groups. Our results also corroborate and extend previous studies, which found that trans people with ethnic minority backgrounds and disabilities face elevated levels of discrimination than other trans people (Kattari et al., 2017; Kattari et al., 2017). Importantly, our study expands this understanding beyond the American context.

### Differences between trans women, trans men, and nonbinary people

When considering differences among trans people, trans women reported experiencing more negative outcomes than trans men, particularly facing higher levels of violence. This is consistent with previous studies showing more discrimination, harassment, and abuse against trans women than trans men (Devís-Devís et al., 2022; Turner & Whittle, 2012). Interestingly, nonbinary people reported discrimination in fewer areas of life than trans women and trans men. This finding diverges from previous studies indicating that nonbinary people generally face higher levels of discrimination than binary trans people (Harrison et al., 2012).

### Strengths and limitations

This study is one of few to employ quantitative analyses to investigate trans people’s experiences in a comparative and intersectional manner. Using recent data from a very large sample across 30 countries in Europe, we applied advanced multilevel modeling techniques to investigate the largely unexplored differences between trans people’s and cisgender LGB people’s experiences. Despite these strengths, the results must be interpreted in light of several limitations.

First, there are limitations regarding the study’s measurement instruments. In particular, experiences of violence relied on a single-item measure, increasing the likelihood that this item inaccurately represents the concept it aims to capture (Cullati et al., 2020). Further, the survey contained inconsistencies that may have resulted in incomplete or skewed representations of some participant’s sexual and romantic attractions. Moreover, the variables for gender and sexuality did not adequately reflect that trans people may identify with a variety of sexualities, including lesbian, gay, and bisexual.

Second, the operationalization of the discrimination variable requires careful consideration. This measure captures the number of distinct life domains (from a set of seven) in which an individual reported experiencing discrimination during the past year. In additional analyses, we examined the presence or absence of discrimination in each single domain (yes/no). We strongly emphasize that this variable measures the breadth of discrimination, not its frequency. It is important to recognize that the relevance of each domain may vary by individual. For example, older adults without children are less likely to interact with school and university settings and thus are less likely to experience discrimination in this context. Further, this measure does not capture the frequency of discriminatory experiences within each domain. As a result, individuals who face persistent or repeated instances of discrimination in one or a few domains may receive lower scores than those who experienced discrimination on isolated occasions across multiple domains.

Third, the measures of disability and ethnic minority status have limitations. Notably, the self-report options provided in these questions were closed-ended, which may restrict participants’ responses. The response options may as such fail to provide a detailed account of potential variability in perceived minority status between participants.

Fourth, our data are not a fully representative sample of Europe’s LGBTQ+ populations because efforts were made to represent a variety of experiences of underrepresented subgroups. As such, while the findings suggest relationships between variables and indicate which groups are more likely to experience discrimination and violence, they do not represent exact prevalence rates for these experiences within each group.

Fifth, tests indicated that data were not missing completely at random. Such missingness patterns may have biased the results; however, only a minimal share of data was missing (0.3%), thereby reducing the likelihood that the bias will substantially impact the results.

Sixth, the intraclass correlations in our multilevel models were low, as was the variance between countries. This likely had an impact on results related to the level-2 predictor, national LGBTQ+ rights. The limited variance across countries may have constrained our ability to detect significant relationships on this level.

Lastly, the original coding for intersex people did not account for those who might also identify as trans before they were excluded from the data. Upon inspecting the data, we found that 727 intersex participants also identified as trans or gender diverse, representing 43.1% of the 1,687 intersex people removed from our analysis. Consequently, this aspect of gender diversity within the trans population was not represented in our analysis. This limitation may affect the demographic profile of the trans population in our sample and could lead to an incomplete understanding of the experiences and needs of the broader trans community, particularly for those who are also intersex.

### Future recommendations

In 2014, the European Union Agency for Fundamental Rights recommended several measures to mitigate discrimination and violence which disproportionately affect trans people across stages and areas of life (FRA, 2014). A decade later, these measures remain highly relevant for current policymaking.

As more data become available, analyses of how trans experiences change over time would be highly useful. This is particularly important given that empirical investigations only recently have attempted to compare trans people’s and cisgender LGB people’s experiences. Building on the present study, future research should examine more closely how national LGBTQ+ rights differentially influence the experiences of trans people and cisgender LGB people. Theoretical and critical explorations of anti-trans movements also deserve more attention to contextualize empirical research. Moreover, future studies should attempt to measure the varying types and intensities of anti-trans mobilization across countries and investigate how these factors predict trans experiences.

When developing interventions that seek to reduce negative experiences and outcomes of LGBTQ+ individuals, it is vital not only to assess overall effectiveness but also to compare their impact on different subgroups. Specifically, outcomes for cisgender LGB people, trans people, and those with intersecting multiple minority identities should be separately evaluated to ensure that interventions are equitable and inclusive. Discrimination and violence are also likely to vary within countries. Therefore, we recommend future studies to investigate city and district-level strata on these dimensions. Moreover, different ethnic minorities are likely to experience varying forms and levels of discrimination and violence. Thus, we also recommend that future research explores the diversity of experiences of LGBTQ+ people across various ethnic backgrounds.

### Conclusion

This study shows that trans people experience more discrimination and violence than cisgender LGB people across 30 European countries. These findings underscore the need to promote institutional and social changes to foster greater acceptance and equality for trans people. Even in countries that promote equality, trans people do not experience improvements to the same degree as cisgender LGB people. This suggests that issues specific to the trans community have yet to be adequately addressed. Our study also highlights the importance of intersectional perspectives. Such approaches offer a comprehensive and critical understanding of how discrimination and violence affect trans people with different nationalities, ethnicities, (dis)abilities, and gender identities.

## Data Availability

For access to data used in this article, please refer to the European Union Agency for Fundamental Rights: https://data.europa.eu/data/datasets/eu-lgbti-ii-survey-experiences-and-views-of-lesbian-gay-bisexual-transgender-and-intersex-lgbti-individuals?locale=en.
